# Case Report: Brucella-associated complications following thoracic endovascular aortic repair: a case series of three patients

**DOI:** 10.3389/fcvm.2026.1885416

**Published:** 2026-10-15

**Authors:** Lei Jing, Linhai Li, Dan Zhou, Weijian Chen, Yonglong Wang, Linguang Han, Chenghui Wang, Xiaojun Shu, Lei Li, Yingxin Tan

**Affiliations:** 1 Jiuquan Hospital of Shanghai General Hospital (Jiuquan People's Hospital), Jiuquan, Gansu, China; 2 Department of Vascular Surgery, General Surgery, The First Hospital of Lanzhou University, Lanzhou, China; 3 The First Clinical Medical School, Lanzhou University, Lanzhou, China; 4 Heart Center, The First Hospital of Lanzhou University, Lanzhou, China; 5 Gansu Provincial Clinical Research Center for Cardiovascular Diseases, The First Hospital of Lanzhou University, Lanzhou, China; 6 School of Clinical Medicine, Tsinghua University, Beijing, China; 7 Vascular Department, Beijing Hua Xin Hospital (1st Hospital of Tsinghua University), Beijing, China

**Keywords:** aortic pseudoaneurysm, aorto-esophageal fistula, brucellosis, endovascular intervention, thoracic endovascular aortic repair

## Abstract

**Background:**

Brucellosis is a rare but potentially fatal cause of vascular graft infection. Evidence regarding Brucella-associated complications after thoracic endovascular aortic repair (TEVAR) remains limited. Case presentation: We report three elderly male patients who developed severe aortic complications following TEVAR, including pseudoaneurysm formation and aorto-esophageal fistula. All patients had a history of epidemiological exposure to Brucella and were diagnosed with active infection through serological testing (Rose Bengal test, serum agglutination test, Brucella IgG and IgM antibodies). Endovascular re-intervention combined with targeted anti-brucellosis therapy was performed in all cases.

**Results:**

One patient achieved sustained clinical remission after repeat endovascular repair and prolonged antimicrobial therapy. Two patients developed catastrophic infectious complications, including mediastinal abscess and aorto-esophageal fistula, resulting in death despite aggressive management.

**Conclusions:**

Brucella infection should be considered in patients presenting with unexplained symptoms after TEVAR, especially in endemic regions. Endovascular repair plays a critical role in acute stabilization; however, long-term outcomes remain highly dependent on effective infection control.

## Introduction

Brucellosis is a zoonotic bacterial infection with a global distribution. Although its cardiovascular complications are rare (approximately 1%–3%) (1), infectious aortic aneurysm—one of its most severe manifestations—progresses rapidly and carries a mortality rate of 21%–22% (2–4). Traditionally, open surgical repair combined with long-term antibiotic therapy has been regarded as the standard treatment; however, it is associated with significant surgical trauma and a high rate of complications. With advances in endovascular techniques, endovascular aortic repair (EVAR/TEVAR) has been increasingly employed in the management of infectious aneurysms due to its minimally invasive advantages. Nevertheless, endovascular repair may be complicated by the development of aortoenteric fistula and pseudoaneurysm, with active Brucella infection representing a rare secondary etiology (5). Therefore, the efficacy and safety of endovascular therapy for infectious aneurysms specifically in the context of Brucella infection require further validation through additional case studies. This article reports three cases of pseudoaneurysm formation following aortic endovascular repair, complicated by concurrent Brucella infection, which were subsequently managed with repeated endovascular intervention.

## Case report

These three cases were encountered consecutively at our institution over the past two years (2022–2023). The diagnosis and treatment of each case were conducted through multidisciplinary team (MDT) discussions, involving vascular surgeons, infectious disease specialists, interventional radiologists, and cardiothoracic surgeons. Management decisions were made collaboratively, taking into account the patients' epidemiological history, clinical presentations, laboratory findings, and imaging features.

The value of a structured MDT approach in managing complex aortic endograft infections has been recently emphasized (6). Multidisciplinary team (MDT) management protocol：All three cases were managed through a structured MDT approach involving vascular surgeons, infectious disease specialists, interventional radiologists, and cardiothoracic surgeons. The decision-making protocol was as follows: Infectious disease specialists performed serological testing and guided anti-Brucella antimicrobial therapy; Interventional radiologists and vascular surgeons jointly reviewed CTA images to assess anatomical suitability for endovascular re-intervention; The entire team collectively weighed the risks and benefits of open versus endovascular repair, considering each patient's hemodynamic status, comorbidities, and infection extent, and reached a consensus on the optimal treatment strategy. This structured approach ensured tailored, patient-specific management for each case.

## Case 1

A 73-year-old male patient was admitted to the hospital due to progressively worsening hemoptysis over three months. The hemoptysis began six months after he underwent thoracic endovascular aortic repair (TEVAR) for a thoracic aortic aneurysm at another institution. The primary procedure was performed on an elective basis according. Following the TEVAR procedure, the patient had experienced persistent chest and back discomfort.

Despite undergoing bronchial artery embolization, coil embolization, and left subclavian artery stenting, the hemoptysis persisted. However, these measures were ultimately ineffective. Although LSA stenting may have targeted collateral supply, it failed to address the primary aortic defect—the persistent endoleak communicating with the pseudoaneurysm cavity. During hospitalization, the patient presented with low-grade fever and reported a history of short-term residence in a pastoral area. Specific infectious disease testing was performed. Serological tests indicated active Brucella infection (positive Rose Bengal test, serum agglutination test +++, positive Brucella IgG and IgM antibodies).

Given the patient's high anesthesia risk and unsuitability for open surgery, he was treated with endovascular intervention. A covered stent was deployed to seal the orifice of the pseudoaneurysm. Given the massive size of the pseudoaneurysm (12.1 cm × 15.7 cm) and the persistence of residual sac perfusion on intra-procedural angiography despite successful proximal stent-graft sealing, we performed adjunctive coil embolization of the residual aneurysm cavity to accelerate intra-sac thrombosis, reduce wall tension, and prevent delayed rupture during the prolonged antimicrobial therapy. The initial TEVAR was performed in Zone 2 (covering the left subclavian artery). Due to pseudoaneurysm formation extending to Zone 1, re-intervention was performed using the chimney technique to reconstruct the left common carotid artery, followed by a reverse branch stent to reconstruct the left subclavian artery, ensuring perfusion of all supra-aortic branches. The left common carotid artery (CCA) was surgically exposed, and its perfusion was maintained using a covered stent placed via the chimney technique. To preserve left subclavian artery flow, a branched endograft component was then deployed from the main aortic stent-graft into the left subclavian artery, establishing a retrograde branch configuration. This approach ensured uninterrupted perfusion to all supra-aortic branches while achieving complete exclusion of the pseudoaneurysm. Additionally, a spring coil was deployed for the embolization of the aneurysm cavity, effectively preventing its expansion.

In the evaluationprior to the emergency re-intervention at our center, no signs of infection were observed. Preoperative physical examinations did not show fever or other signs of infection, and blood tests revealed no elevated infection markers such as white blood cell count or C-reactive protein. Additionally, preoperative imaging (CT and CTA) did not reveal any infection-related abnormalities, such as arterial wall thickening or features of a pseudoaneurysm. Therefore, there were no signs of infection at the time of the first TEVAR procedure in these cases.

Concurrently, standard anti-Brucella antimicrobial therapy was initiated. One month later, the patient was readmitted due to fever. A chest CT scan revealed mediastinal emphysema, and CT-guided drainage yielded foul-smelling brownish fluid (Figure 1). Bacterial culture of the purulent fluid grew Escherichia coli. The decision to perform percutaneous rather than surgical drainage was made after MDT discussion, considering the patient's recent repeat endovascular intervention, advanced age with high anesthetic risk, and the presence of an aortic stent-graft in the infected field, where surgical exploration carried substantial risk of graft disruption or catastrophic hemorrhage. Although his body temperature normalized after drainage, the patient died suddenly on the fourth day post-drainage. The cause of death was likely severe infection.

**Figure 1:**
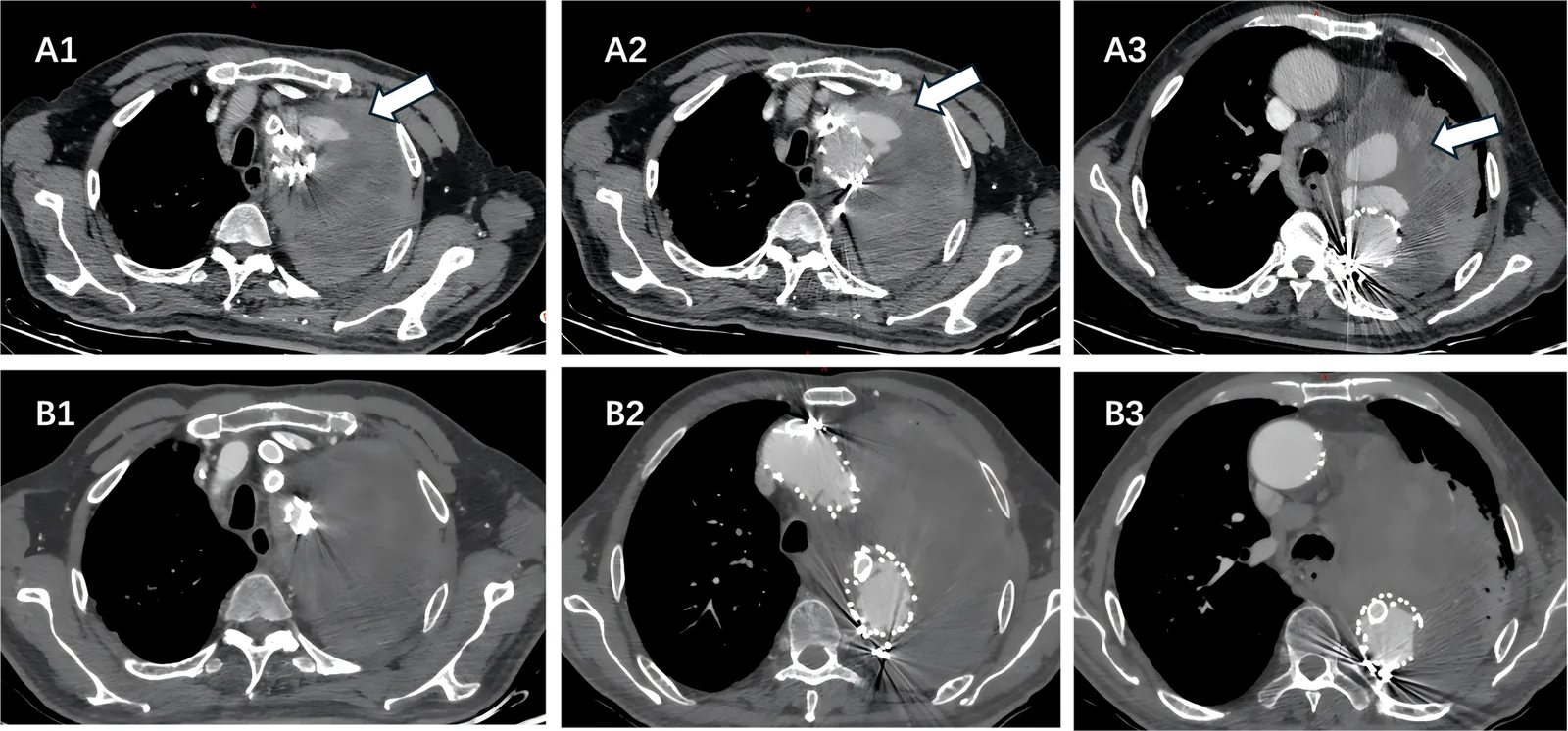
Comparison of pre- and post-operative CT angiography (CTA). **A**. Pre-operative CTA: Multiple recesses (or ulcerations) are observed along the outer contour of the aortic arch stent-graft. The arrow indicates an area of endoleak, showing heterogeneous attenuation within. The lesion measures 12.1 cm  ×  15.7 cm. B. Post-operative CTA (10-day follow-up): Follow-up CTA shows resolution of the contrast endoleak. There is satisfactory contrast opacification within the stent-graft and in the distal aortic segment.

## Case 2

A 69-year-old male patient presented with a two-week history of intermittent chest pain accompanied by fever. He had a history of thoracic endovascular aortic repair (TEVAR) performed at another hospital 10 months prior for a thoracic aortic aneurysm.

Upon admission, aortic CT angiography (CTA) showed no significant abnormalities. Serological tests confirmed Brucella infection (positive Rose Bengal test, serum agglutination test ++, positive Brucella IgG and IgM antibodies). After initiating standardized anti-Brucella antibiotic therapy, the patient's chest pain and fever showed improvement. However, on the fourth day of antimicrobial treatment, he experienced massive hematemesis (approximately 1,500 mL) leading to hemorrhagic shock. Emergency repeat CTA revealed the formation of a pseudoaneurysm in the aortic arch (Figure 2). Subsequent gastroscopy confirmed an esophageal fistula, demonstrating fistula-like changes with visible blood clots on the surface and active bleeding at the margins (Figure 3). Upon admission, immediate volume resuscitation with blood transfusion and acid suppression therapy were initiated for hemorrhagic shock. No therapeutic endoscopic intervention was attempted; after diagnosis confirmation, the patient was transferred directly for emergency TEVAR.

**Figure 2:**
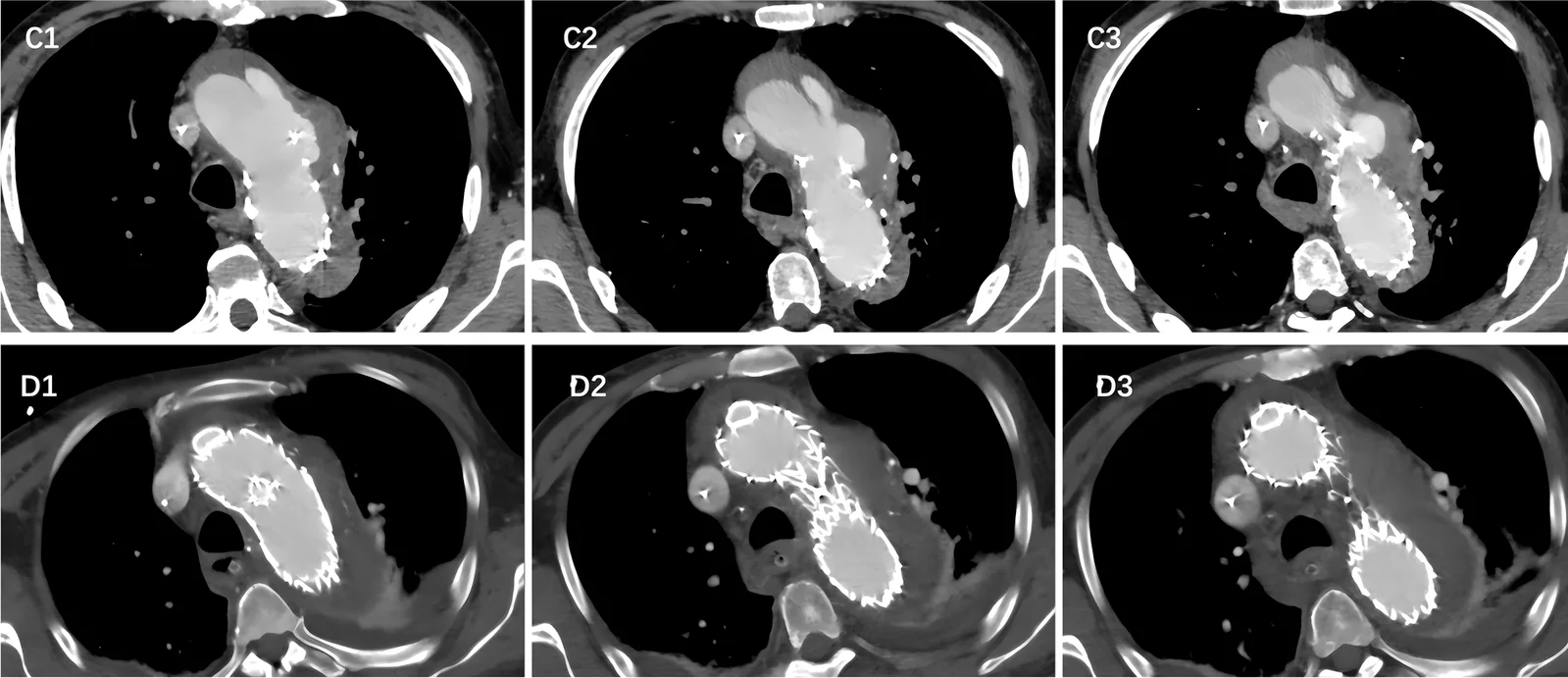
Comparison of Pre- and post-procedural CT angiography (CTA). **C**. CT angiography reveals a pseudoaneurysm of the aortic arch, which developed following a thoracic endovascular aortic repair (TEVAR) procedure performed 10 months prior. **D**. Post-procedural CTA after endovascular exclusion of the pseudoaneurysm.

**Figure 3:**
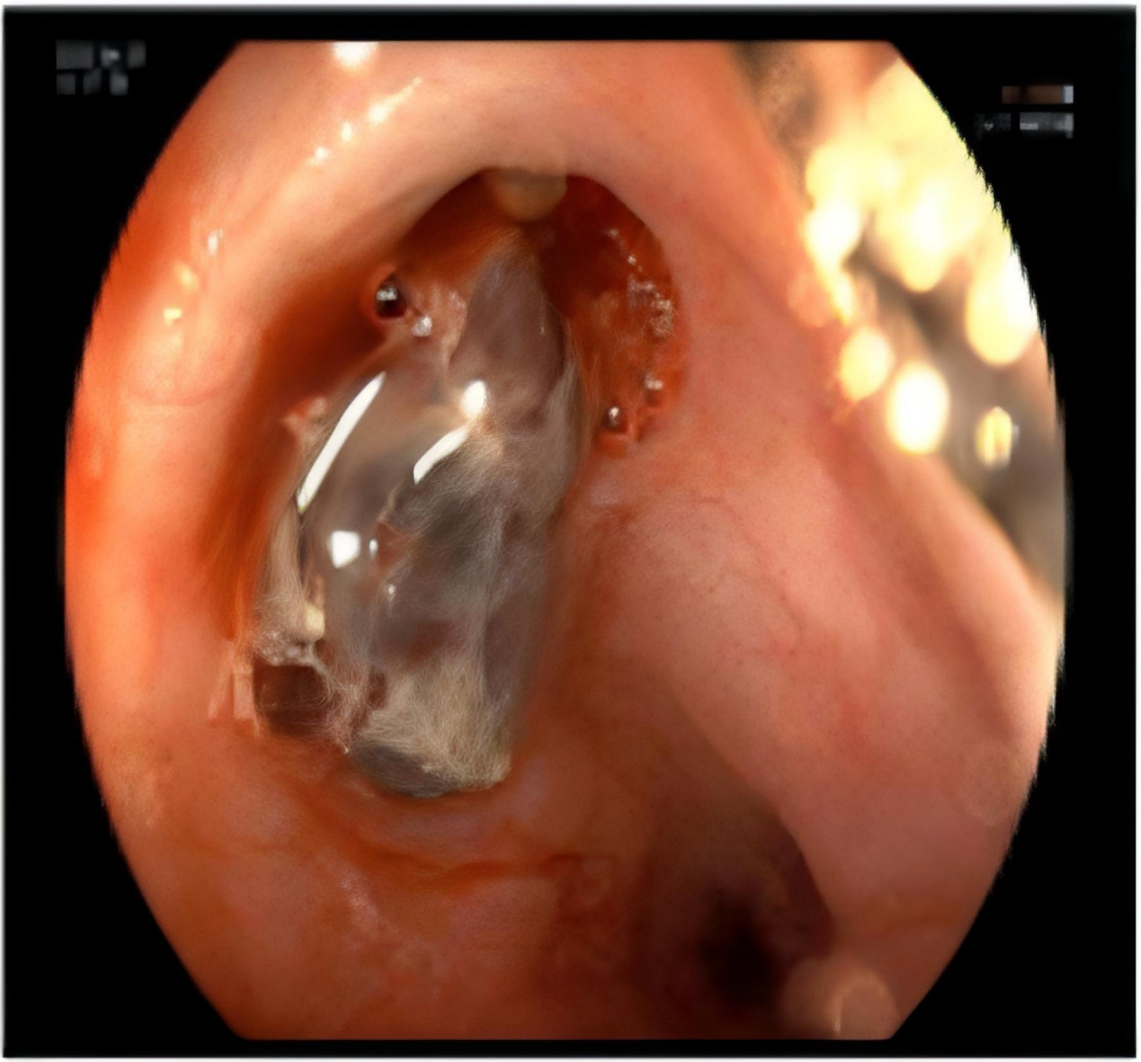
Esophagogastroscopy reveals a fistula-like lesion located 33 cm from the incisors, with a surface covered by blood clots and active bleeding at the margins.

Given the patient'sactive hemorrhagic shock (1,500 mL hematemesis), which precluded tolerance of single-lung ventilation and aortic cross-clamping, combined with the severely infected local tissue (aorto-esophageal fistula) that would have required extensive debridement and carried an unacceptably high risk of intraoperative bacteremic spread and graft reinfection, open surgical conversion was deemed contraindicated. Consequently, endovascular exclusion of the pseudoaneurysm was selected as a rapid, minimally invasive, and life-saving strategy to achieve immediate hemorrhage control, with the understanding that definitive infection control would rely on prolonged antimicrobial therapy (Figure 2). The pseudoaneurysm was located in Zone 2. During emergency re-intervention, the landing zone was extended to Zone 0. The chimney technique was used to reconstruct the brachiocephalic trunk, left common carotid artery, and left subclavian artery. A stent was anchored at the Z0 zone, and the brachiocephalic artery was reconstructed using the chimney stent technique. Both the left common carotid artery (CCA) and the left subclavian artery were reconstructed using the *in-situ* fenestration, in which a puncture needle was used to create fenestrations in the stent-graft, followed by covered stent implantation. This surgical strategy successfully restored blood flow while ensuring the patency of the critical arteries. Postoperatively, standardized anti-Brucella treatment was continued. Unfortunately, the patient succumbed to a subsequent episode of hematemesis over two months after the procedure.

## Case 3

An elderly male was admitted to our center due to a three-day history of hemoptysis. Seven months prior to this admission, he had undergone TEVAR at an outside hospital for a pseudoaneurysm, reportedly on an elective basis. Baseline aortic diameters and stent-graft details were not available from the outside records, and serological testing for Brucella had not been performed at that time.

Early post-operative follow-up(performed at the outside institution) demonstrated a well-positioned aortic stent-graft with satisfactory morphology and no signs of endoleak or migration on CT angiography (CTA) (Figure 4).

**Figure 4:**
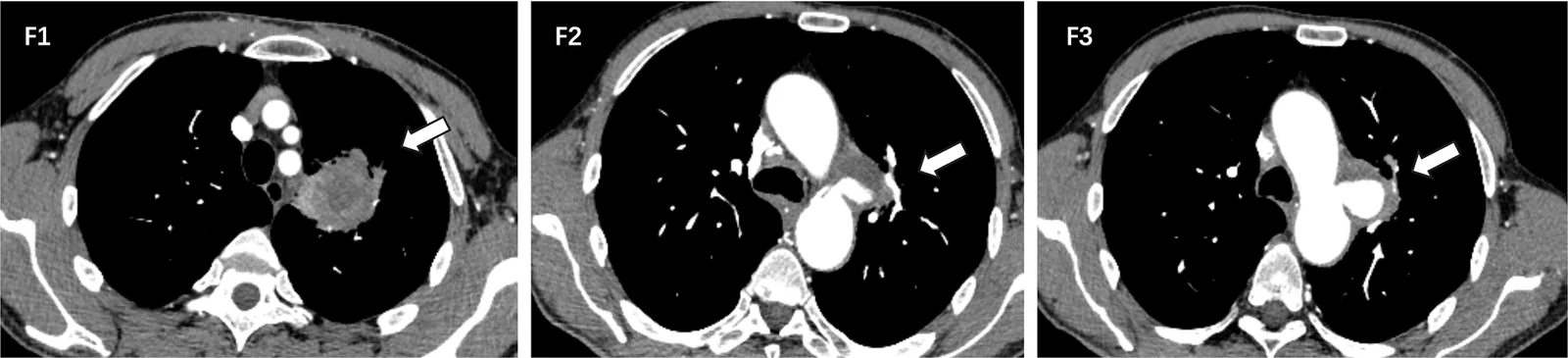
The patient's first pre-operative CTA demonstrates a pseudoaneurysm. Brucellosis was not diagnosed at that time.

At the time of current presentation to our center, contrast-enhanced aortic CT revealed a new pseudoaneurysm formation distal to the thoracic aortic stent-graft, accompanied by a mediastinal hematoma (Figure 5). Given the patient's history of cattle and sheep exposure, further serological testing was performed and confirmed active Brucella infection (positive Rose Bengal test, serum agglutination test ++++, positive Brucella IgG and IgM antibodies).

**Figure 5:**
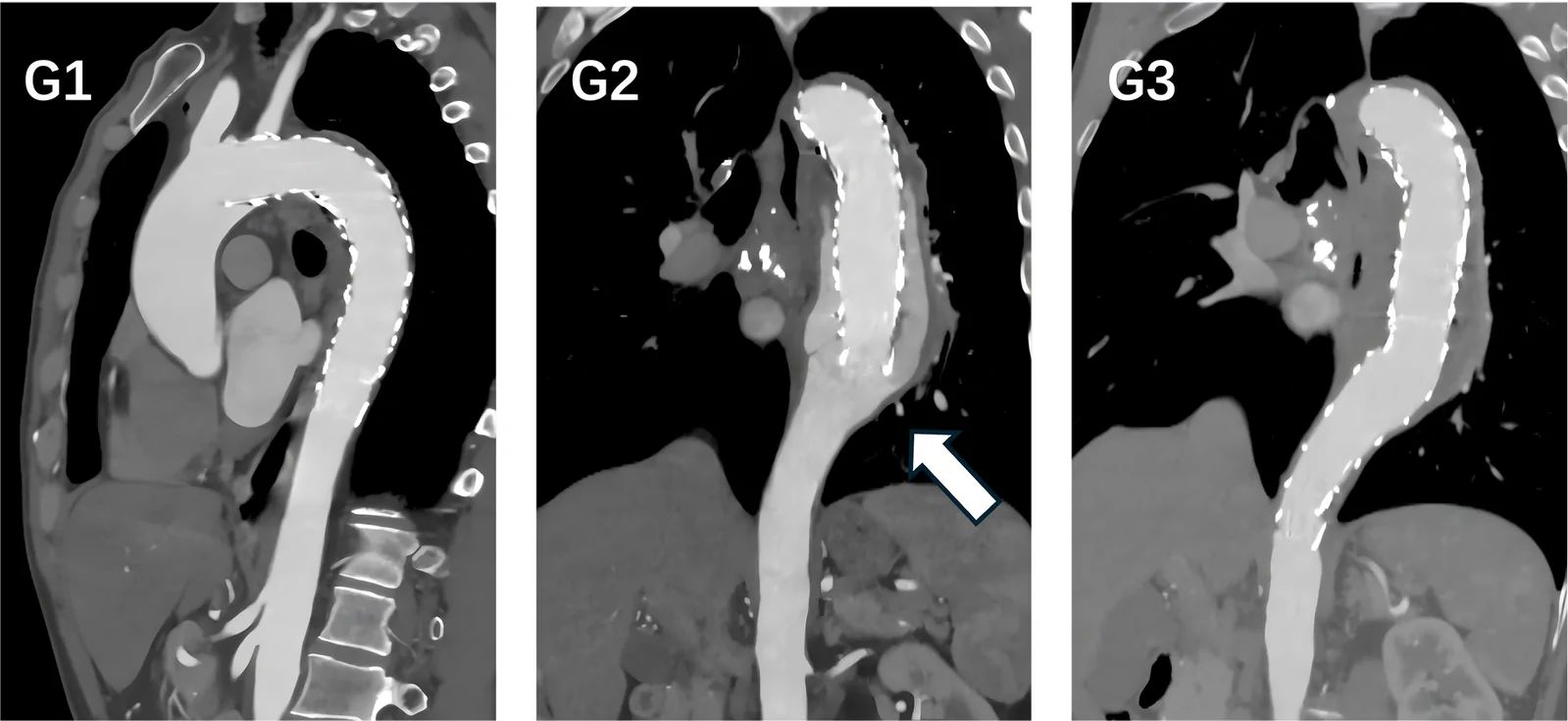
The early post-operative CTA follow-up after the initial procedure demonstrates satisfactory morphology and correct position of the aortic stent-graft, with no signs of endoleak or migration noted (G1). The CTA upon current hospital admission reveals the formation of a new pseudoaneurysm distal to the thoracic aortic stent-graft, accompanied by a mediastinal hematoma (G2). Following repeat endovascular repair combined with long-term anti-Brucellatherapy, a subsequent CTA shows successful exclusion of the pseudoaneurysm and restoration of stable stent-graft configuration.

As the family declined open surgical intervention, the patient underwent a repeat endovascular exclusion procedure (Figure 5). The pseudoaneurysm was located in the distal descending thoracic aorta. Re-intervention involved implanting a new covered stent distal to the original stent-graft to completely exclude the lesion. This was followed by standardized anti-Brucella antimicrobial therapy.

The patient is a 59-year-old male with pre-existing comorbidities including hypertension, hepatitis B, and a history of colon cancer. Notably, he had been diagnosed with Brucella infection at a local hospitalone year prior to the current presentation and had completed a 6-month course of anti-brucellosis therapy at that time; however, serological testing upon admission to our center still confirmed active infection, suggesting either relapse or persistent disease.

Following the repeat endovascular intervention, the patient has been followed for 12 months and remains generally well, without new aortic complications or recurrence of hemoptysis. Our surveillance protocol includes: clinical assessment with symptom review and physical examination every 3 months during the first year; serological testing (Rose Bengal test and serum agglutination test) at each visit to monitor for relapse; and annual aortic CTA to assess stent-graft integrity, detect new pseudoaneurysm formation, or identify any structural changes. To date, no abnormalities have been detected.

## Antimicrobial management

Following serological confirmation of active Brucella infection, all three patients received a standardized and prolonged antimicrobial regimen, which was initiated immediately after diagnosis and continued throughout the follow-up period. The regimen consisted of: (1) Rifampicin capsules, 0.9 g (6 capsules), orally, once daily (qd); (2) an additional single dose of Rifampicin 0.9 g (6 capsules), orally; and (3) Doxycycline hyalclate enteric-coated capsules, 0.1 g, orally, twice daily (bid). This combination was prescribed according to the World Health Organization (WHO) guidelines and local consensus for the treatment of brucellosis. All doses and frequencies were administered as prescribed.

## Discussion

Brucellosis is a zoonotic infection whose causative pathogen exhibits significant endothelial and vascular tropism. In the present series, the diagnosis of aortic graft infection was systematically evaluated according to the Management of Aortic Graft Infection Collaboration (MAGIC) criteria (7). Case 1 and Case 2 met the clinical/surgical major criteria (aorto-bronchial and aorto-esophageal fistula, respectively), while Case 3 met the radiological major criterion (new perigraft pseudoaneurysm). All three cases were further supported by laboratory minor criteria (positive Brucellaserology). Therefore, all patients in this report were classified as having definite aortic graft infections, which underscores the critical role of Brucella as a causative pathogen in post-TEVAR complications. The rapid progression to pseudoaneurysm and fistula within 6–10 months post-TEVAR, combined with uniformly positive Brucella serology, suggests that the primary aortic pathology was most likely an initially unrecognized mycotic aneurysm rather than a standard degenerative lesion. The stent-graft may have further exacerbated local inflammation by serving as a nidus for bacterial adherence. While its cardiovascular complications occur at a low incidence, they represent a major cause of mortality associated with brucellosis. Aortic involvement is particularly rare and severe. Notably, in patients with pre-existing vascular stent grafts, biofilm formation and chronic inflammatory responses during the recovery process can significantly increase the risk of graft infection (8). The risk of endograft infection after complex aortic repair has been recently characterized (9). In the present series, all three patients presented with active Brucella infection and severe post-TEVAR complications—including uncontrollable hemoptysis, hemorrhagic shock, and acute pseudoaneurysm rendering them unsuitable for open surgical repair due to prohibitive risk (10). Their high-risk status was multifactorial:1. physiological—advanced age (>50 years), high anesthetic risk in Case 1, and ongoing hemorrhagic shock in Case 2 precluding tolerance of single-lung ventilation and aortic clamping (11), 2. infectious—open repair would require extensive debridement and prosthetic grafting, with extreme risks of intraoperative bacteremic spread and subsequent graft infection (11); 3. Upon evaluation, the anatomical conditions of the lesion are suitable for endovascular surgery, allowing rapid endovascular coverage to achieve emergency hemostasis before fatal hemorrhage occurs (12). Thus, despite the inherent risks of late reintervention and persistent infection, emergency endovascular repair (13)combined with long-term antibiotics represented the only viable strategy (10) to balance immediate survival and long-term prognosis in this cohort.

The diagnostic and therapeutic journey of this case series highlights current challenges in managing brucella-related aortic disease. Due to its insidious clinical course and non-specific symptoms, it is often misattributed to post-operative complications or general bleeding rather than infection, leading to underdiagnosis. Therefore, in endemic regions, a high index of suspicion for Brucella infection should be maintained in patients presenting with unexplained fever, bleeding, or pain following endovascular repair. Definitive diagnosis relies on epidemiological history and serological testing.

The European Society for Vascular Surgery (ESVS) guidelines indicate that the standard treatment for infectious aortic pathologies is open surgical repair combined with long-term antibiotic therapy (14).However, elderly patients and those with multiple comorbidities often cannot tolerate open surgery.

Since Kusztal's first report in 2007 on using TEVAR to treat a brucella-related aortic aneurysm (15), subsequent studies, such as Benedetto et al.'s successful management of an aortic rupture case in 2011 using emergency EVAR combined with long-term antibiotics (16), have supported the model of endovascular repair combined with prolonged antimicrobial therapy. Existing data show a 5-year survival rate of 55% for endovascular aortic aneurysm repair combined with long-term antibiotic therapy (17), further substantiating the feasibility of this strategy. The cases presented in this series demonstrate that endovascular repair holds irreplaceable value in controlling acute hemorrhage and preventing rupture. However, it does not eradicate the infection *per se* and should be viewed more as part of a comprehensive infection control strategy or a bridging measure.

Aorto-esophageal fistula is one of the most lethal complications following TEVAR. When an aneurysm compresses the adjacent esophagus leading to fistula formation, it can trigger fatal upper gastrointestinal hemorrhage, which is particularly perilous in the setting of co-existing infection. Clinically, approximately 45% of patients present with the classic ‘Chiari's triad' – chest pain, sentinel hemorrhage, and ultimately massive hematemesis (18). Case 2 exhibited this pattern of prodromal symptoms followed by catastrophic bleeding. Studies indicate that for patients with aorto-esophageal fistula, if conservative antibiotic treatment is chosen, the chance of survival is extremely low, with mortality reaching 100% within days (19, 20).Even with timely endovascular exclusion combined with anti-infective therapy, the long-term prognosis remains poor. Persistent infection and nutritional impairment are significant contributors to late mortality.

Reviewing these three cases, this series suggests that: firstly, in endemic areas, a high suspicion for brucellosis should be maintained when unexplained symptoms arise after TEVAR; secondly, while endovascular therapy is crucial in emergency management, it must be combined with standardized, long-term anti-Brucella treatment; thirdly, even with aggressive treatment, patient long-term outcomes remain suboptimal, underscoring the importance of early diagnosis and multidisciplinary collaboration. Although endovascular surgery combined with pharmacotherapy has proven feasible, how to effectively prevent and manage long-term post-procedural complications requires further clinical research. This case series provides valuable practical insights in this regard.

Furthermore, given the indolent nature of Brucella infection and the risk of late recurrence, long-term follow-up is mandatory in these patients. While CTA remains essential for assessing structural integrity and detecting morphological changes such as pseudoaneurysm formation or stent-graft migration, it has limited sensitivity for detecting low-grade infection within the graft. Therefore, we recommend incorporating18F-FDG-PET/CTinto the surveillance protocol due to its high sensitivity for detecting residual or recurrent metabolic activity at the graft site (21). This is particularly valuable after the completion of antibiotic therapy (e.g.,at 6–12 months) or when there is clinical suspicion of relapse despite negative CTA findings. A multidisciplinary approach combining clinical assessment, serological testing, and advanced imaging is crucial for optimizing long-term outcomes in this challenging patient population.

Endovascular repair in this series served primarily as a bridging tool to achieve immediate hemodynamic stabilization rather than a definitive cure. We fully acknowledge that placing additional prosthetic material (covered stents and coils) in an actively infected field carries a theoretical risk of persistent infection or prosthetic colonization. However, in our emergency cases, this risk was outweighed by the immediate life-threatening hemorrhage, for which endovascular repair was the only viable option. More aggressive hybrid strategies—including surgical fistula closure, saccotomy with graft preservation, or omental flap coverage—were not feasible due to hemorrhagic shock and prohibitive surgical risk in our patients.

## Limitations

This retrospective study includes only three cases; therefore, the current conclusions lack strong supporting evidence. Larger-scale prospective clinical studies are needed to further validate the long-term efficacy, safety, and outcomes of endovascular treatment for brucella-related aortic aneurysms.

Due to the rarity of the condition and the fact that long-term outcomes are not yet fully understood, endovascular treatment should currently be regarded as a temporary bridging strategy, particularly in acute and life-threatening settings, rather than a definitive long-term solution. Nonetheless, when combined with standardized and prolonged antimicrobial therapy, endovascular repair may offer a safe and effective option for managing aortic pseudoaneurysms and related complications secondary to Brucella infection, especially in patients who are poor candidates for open surgery.

Additionally, 18F-FDG-PET/CT—a highly sensitive imaging modality for detecting graft infection—was not utilized in this series due to its retrospective design and the unavailability of this technique during follow-up. Future large-scale prospective studies incorporating advanced imaging modalities and extended surveillance are warranted to better evaluate the long-term efficacy and safety of endovascular therapy in this context.

Detailed baseline data for the primary TEVAR procedures (aortic diameters, graft specifications, and initial aneurysm morphology) were unavailable as all index surgeries were performed at external institutions.
